# Sperm Antioxidant Capacity Discriminates Between Fertile and Infertile Men and Is Strictly Related to Lipid Peroxidation and Lipid Mediator Production

**DOI:** 10.3390/biology15100760

**Published:** 2026-05-10

**Authors:** Cinzia Signorini, Elena Moretti, Laura Liguori, Elena Leoni, Caterina Marcucci, Maria Cristina Salvatici, Giulia Collodel

**Affiliations:** 1 Department of Molecular and Developmental Medicine, University of Siena, 53100 Siena, Italy; cinzia.signorini@unisi.it (C.S.); laura.liguori@student.unisi.it (L.L.); elena.leoni@student.unisi.it (E.L.); caterin.marcucci@student.unisi.it (C.M.); giulia.collodel@unisi.it (G.C.); 2 Centro di Microscopie Elettroniche “Laura Bonzi”, Institute of Chemistry of Organometallic Compounds (ICCOM), Consiglio Nazionale delle Ricerche (CNR), 50125 Firenze, Italy; salvatici@ceme.fi.cnr.it

**Keywords:** antioxidants, F_2_-Isoprostanes, human spermatozoa, inflammation, oxidative stress, Resolvin D1, ROC curve, Trolox equivalent antioxidant capacity, TEM

## Abstract

Oxidative stress (OS) and inflammation are common causes of male infertility; antioxidants in spermatozoa and especially in seminal plasma play a protective role. The study aims to evaluate whether OS lipid mediators (F_2_-isoprostanes: F_2_-IsoPs; Resolvin D1: RvD1) measured in seminal plasma, and Trolox equivalent antioxidant capacity (TEAC), measured in both seminal plasma and spermatozoa, are associated with male infertility. Semen samples (18 fertile and 62 infertile subjects) were examined following WHO guidelines and with a mathematically elaborated transmission electron microscopy analysis (fertility index-FI-, % sperm pathologies). F_2_-Isops were measured by GC/NICI-MS/MS, RvD1 by ELISA, and TEAC by a commercially available antioxidant assay. F_2_-Isops, RvD1, and sperm TEAC (positively interrelated with each other) correlated negatively with seminal parameters and FI and positively with sperm pathologies. F_2_-Isops, RvD1, and sperm TEAC levels were significantly higher in infertile vs fertile subjects (*p* < 0.001). ROC curve analysis demonstrated that sperm TEAC (J index: 0.13 mM) and seminal RvD1 (J index: 38.26 pg/mL) discriminated between fertile and infertile subjects. Spermatozoa stimulate antioxidant capacity in the presence of an OS environment; this data suggests an association in which antioxidant defences may vary in relation to the surrounding seminal plasma. Moreover, sperm TEAC, and to a lesser extent seminal RvD1, emerge as potential markers for identifying infertile patients.

## Simple Summary

Male infertility is often linked to damage caused by harmful molecules that cause oxidative stress (OS) and inflammation. Human semen has natural defences, called antioxidants, present in spermatozoa and seminal plasma that help protect against this damage. This study aimed to evaluate the relationships among F_2_-isoprostanes (F_2_-IsoPs, OS marker), Resolvin D1 (RvD1, index of resolution of inflammation), and total antioxidant capacity, expressed as Trolox equivalent antioxidant capacity (TEAC), measured in spermatozoa and seminal plasma of infertile and fertile subjects. Semen parameters and levels of these compounds were compared. We found that infertile men had higher levels of damage-related markers in seminal plasma and increased antioxidant capacity in spermatozoa compared to fertile men. Further analysis showed that sperm TEAC and seminal RvD1 were able to effectively distinguish between fertile and infertile men. These findings suggest that sperm actively respond to stress by increasing their protective defences. Moreover, TEAC in sperm and RvD1 in seminal plasma, together with F_2_-IsoPs (previously demonstrated), may serve as potential markers for identifying male infertility.

## 1. Introduction

Oxidative damage to lipids in sperm membranes and inflammatory processes are events recognized to be relevant in determining the quality of human semen [1,2,3]. Antioxidant defence plays a key role in the molecular mechanisms involved in these pathological mechanisms and is increasingly studied as a protective factor during the semen manipulation phases. The ability to hinder the activity of oxidants, including free radical species (reactive oxygen species, ROS), is a relevant topic in biology, and increasing possibilities for enhancing this defensive potential are studied. In fact, the amount and effects of ROS must be regulated and adequately balanced by the action of antioxidants, both enzymatic and non-enzymatic, to avoid excessive oxidative action mediated by oxidants.

In human semen, the relevance of the balance between the production of ROS and antioxidant defences is well established [4]. It is known that sperm function requires the presence and action of free radicals; in spermatozoa, ROS represent a metabolic product of aerobic processes. Within limited levels, ROS play a key role in signal transduction pathways and sperm physiology. In particular, ROS are involved in the regulation of motility, capacitation, acrosome reaction, and interaction with oocytes [5,6]. Conversely, the accumulation of oxidants determines an advantage of oxidative actions over antioxidant ones, thus creating the conditions for oxidative damage of sperm cell structures [3]. Nevertheless, it is important to specify that, in biological systems, the oxidants acting as physiological messengers in redox signalling and the oxidants responsible for the oxidative damage of biomolecules are not the same [7,8].

The sperm membrane, being particularly rich in polyunsaturated fatty acids (PUFA), represents a particularly susceptible target to oxidative-mediated damage, triggering the lipid peroxidation (LPO) cascade. Secondary products of LPO are recognized as biomarkers of oxidative-mediated lipid oxidation. Among these, F_2_-isoprostanes (F_2_-IsoPs), which are prostaglandin-like compounds, are widely used in the quantification of non-enzymatic oxidation of arachidonic acid, the fatty acid precursor of F_2_-IsoPs. In particular, the detection of F_2_-IsoPs, which has been performed in different biological samples, is recognized as a gold standard method to evaluate oxidative stress (OS) in different conditions [9,10,11,12,13,14,15,16,17].

In 2022, our research group established a seminal plasma F_2_-IsoP cut-off value of 29.96 ng/mL. This threshold effectively discriminates fertile men, characterized by low LPO levels, from infertile patients with elevated LPO levels [18]. For a comprehensive evaluation of the role of LPO in influencing sperm quality and male infertility, it is important to examine both the biomarkers of PUFA oxidative damage and the antioxidant capacity available to counteract OS. The antioxidant capacity may be expressed as the Trolox equivalent antioxidant capacity (TEAC), both in cellular extract and media [19,20].

Furthermore, the role of inflammation, even in its subclinical form, is relevant for the quality and functionality of sperm. In this view, a prominent role in regulating inflammatory events is carried out by the inflammatory mediators specialized in promoting inflammation resolution (SPMs), acting as signalling molecules able to drive the resolution of inflammation. Among SPMs, resolvins, which are derived from eicosapentaenoic (EPA, 20:5n-3) and docosahexaenoic (DHA, 22:6n-3) fatty acids—in particular, Resolvin D1 (RvD1), whose DHA is the fatty acid precursor—exert anti-inflammatory and pro-resolution activity in acute inflammation [21].

In the assessment of sperm quality, the semen is first analyzed by performing a spermiogram (semen analysis) as defined by WHO guidelines. However, an in-depth morphological assessment of spermatozoa is only possible through transmission electron microscopy (TEM); this technique allows for the identification of defects in individual organelles and their related pathologies, enabling a clear diagnosis of systematic versus non-systematic defects [22]. Using this methodology, Baccetti and colleagues defined a formula capable of expressing the percentage of pathologies such as apoptosis, immaturity, and necrosis, as well as a fertility index for each sample [23,24].

To understand oxidative damage in human spermatozoa, semen parameters are evaluated—specifically sperm motility—as well as its presence and association with pathological conditions detected with different methods [25,26,27]. Malondialdehyde (MDA) and F_2_-IsoPs have been established as markers of seminal oxidative damage [28,29], while resolvins, in particular RvD1, act as a mediator of inflammation resolution in semen [30].

The aim of this study is to evaluate whether lipid biomarkers (F_2_-Isops and RvD1) of OS measured in seminal plasma, and TEAC, measured in both seminal plasma and spermatozoa, are associated with male fertility status. To this end, semen samples obtained from fertile men and infertile patients were both examined following WHO guidelines [31] and with a mathematically elaborated TEM analysis. Then, the final goal was to develop robust indices capable of accurately discriminating between fertile and infertile individuals.

## 2. Materials and Methods

### 2.1. Subjects

In this partially retrospective study (2015–2025), we enrolled 62 infertile Italian male subjects (aged 22–39) who sought consultation at the Department of Molecular and Developmental Medicine after failing to achieve pregnancy following at least one year of attempting to conceive [31,32]. A control group of 18 fertile men, aged 22–37 years, (BMI ≤ 25), all of whom had fathered at least one child in the previous three years and without signs of infection or anatomical abnormalities, was also enrolled.

For the patients and controls selected for the study, aliquots of seminal plasma and spermatozoa from the same sample on which TEM had been performed were stored at −80 °C. The samples were thawed only once, and therefore we do not have the bias associated with multiple freeze–thaw cycles.

Patients were non-azoospermic, non-paraplegic, and non-obese (BMI ≤ 25), and had no history of radiotherapy, chemotherapy, chronic illness, medication use, testicular cancer, or drug use. None of the men showed known systematic sperm defects. None of them took an oral antioxidant supplement for 6 months before the semen analysis. The female factor was excluded as reported by medical history and gynecological evaluation.

At the time of the analysis, patients and controls provided written informed consent for inclusion in the Centre’s research, according to the guidelines of the period for respecting privacy and the Helsinki Declaration of 1975, or in accordance with the requirements of the Ethics Committee of Siena University Hospital [CEAOUS ID: CEAVSE 25612]. Patients were assured that their semen samples would be used exclusively for the approved research protocol and were strictly excluded from use in any assisted reproductive technology (ART) procedures.

**Figure 1:**
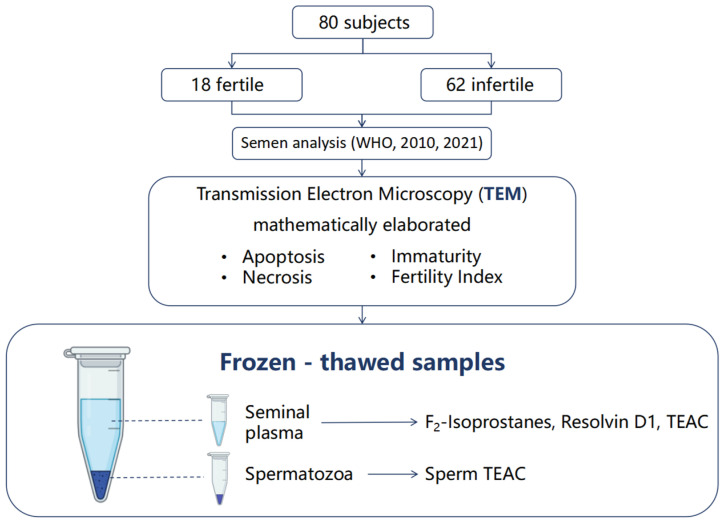
Study design: a total of 80 semen samples were analyzed following WHO guidelines (2010, 2021) [31,32], and, by TEM analysis, mathematically elaborated [23]. The frozen seminal plasma was used to measure F_2_-isoprostanes, Resolvin D1 and Trolox equivalent antioxidant capacity (TEAC); TEAC was also assayed in frozen spermatozoa.

The study design is reported in Figure 1.

### 2.2. Light Microscopy

Semen samples were collected by masturbation after 3–5 days of sexual abstinence and examined after liquefaction for 30 min at 37 °C. Volume, pH, sperm concentration, and motility were assessed as recommended by World Health Organization guidelines [31,32]. In samples analyzed by the laboratory before 2021, the sperm motility was evaluated as rapid and slow [32], and in the cases after 2021, as sperm progressive motility (recommended by the WHO [31]). The evaluation of sperm morphology was performed using the stain-coated Testsimplets slides (Origio, Firenze, Italy).

### 2.3. Transmission Electron Microscopy

For the TEM procedure, sperm samples were fixed in cold Karnovsky fixative and maintained at 4 °C for 2 h. Then, the semen was washed in 0.1 mol/L cacodylate buffer (pH 7.2) for 12 h, postfixed in 1% buffered osmium tetroxide for 1 h at 4 °C, and washed again in 0.1 mol/L cacodylate buffer. The samples were dehydrated in a graded ethanol series and embedded in Epon Araldite. Ultra-thin sections were cut with a Supernova ultramicrotome (Reickert Jung, Vienna, Austria), mounted on copper grids, stained with uranyl acetate and lead citrate, and then observed and photographed with a Philips CM10 and Philips CM12 transmission electron microscopes (TEM; Philips Scientifics, Eindhoven, The Netherlands, Centro di Microscopie Elettroniche “Laura Bonzi”, ICCOM, Consiglio Nazionale delle Ricerche –CNR-, Via Madonna del Piano, 10 Firenze, Italy).

Three hundred longitudinal and cross-sections of sperm were examined for each sample, and the assessments were performed by an operator blinded to group allocation. The TEM data obtained was processed using a Bayesian method used in our laboratory for 30 years [23]. The model incorporates, as input parameters, the numbers of a comprehensive set of ultrastructural defects observed in the different sperm sections. Specifically, the evaluated variables include defects affecting: acrosome (position, dimension, shape, content), shape of the nucleus (normal, roundish, altered), chromatin texture (condensed, immature, necrotic, with vacuoles), mitochondria (shape and organization), axonemal structures (9 + 2 arrangement, dynein arms), periaxonemal components (outer dense fibres, fibrous sheath), plasma membrane integrity, and the presence or absence of cytoplasmic residues.

From these inputs, the method produces as outputs: (a) the fertility index (FI) expressing the number of defect-free spermatozoa; and (b) the percentage of spermatozoa with immaturity, necrosis and apoptosis [24]. These sperm pathologies are well defined by peculiar ultrastructural alterations: necrotic sperm show reacted/absent acrosome, altered nuclei with disrupted chromatin texture, damaged plasma membrane, and poor axonemal cytoskeletal structures. Apoptotic sperm have chromatin, which tends to be segregated along the margin of the nuclear membrane, the presence of cytoplasmic residues, and swollen and disorganized mitochondria. The presence of spherical and elliptical nuclei with uncondensed chromatin, altered acrosomes, and cytoplasmic droplets are markers of immature sperm.

### 2.4. Seminal F_2_-Isoprostane Determination

To perform the quantification of F_2_-IsoPs, a basic hydrolysis was carried out by adding 1 N KOH (incubation at 45 °C for 45 min). Subsequently, 1 N HCl and 500 pg Prostaglandin F_2α_-d_4_ were added, before carrying out the subsequent sample purification by a solid phase extraction procedure. In the purification step, each sample was first applied to an octadecylsilane (C_18_) cartridge (500 mg Cartridge, 55–105 µm Particle Size, 6cc, Waters, Milford, MA, USA), and subsequently, to an aminopropyl (NH_2_) cartridge (500 mg Cartridge, 55–105 µm Particle Size, 6cc, Waters, USA). In each purified sample, a derivatization step was carried out and then the spectrometric analysis was performed. In the gas chromatography/negative ion chemical ionization tandem mass spectrometry (GC/NICI-MS/MS) (Thermo Finnigan, San Jose, CA, USA, instrumentation), F_2_-IsoPs were quantified by identification of the *m*/*z* 299 ion produced by ionization of 8-Isoprostane, the most represented F_2_-IsoP isomer [29]. The amount of 8-Isoprostane was determined by comparing the ratio of 8-Isoprostane (No. 16350, Cayman Chemical, Ann Arbor, MI, USA) to Prostaglandin F_2α_-d_4_ (Cayman Chemical, Item No. 316010), against a standard calibration curve.

### 2.5. Seminal Resolvin D1 Evaluation

Semen RvD1 levels were quantified using a sandwich enzyme-linked immunosorbent assay (ELISA) kit (MBS2601295-96, MyBioSource, San Diego, CA, USA). The assay employed a biotin-labelled antibody and a horseradish peroxidase (HRP)-avidin system for detection. Absorbance was measured spectrophotometrically at 450 nm, and RvD1 concentrations were determined by comparing the optical density of each sample against a standard curve (range: 31.2–2000 pg/mL).

### 2.6. Antioxidant Capacity Evaluation

In spermatozoa and seminal plasma of each sample, the antioxidant capacity was measured as the ability of sample antioxidant compounds to scavenge a radical cation compared to the standard antioxidant, the water-soluble vitamin E analogue (±)-6-Hydroxy-2,5,7,8-tetramethylchromane-2-carboxylic acid (Trolox). Thus, the antioxidant capacity was evaluated as the Trolox equivalent antioxidant capacity (TEAC). In particular, the principle of the antioxidant assay applied is the formation of a ferryl myoglobin radical from metmyoglobin and hydrogen peroxide, which oxidizes the 2,2′-azino-bis (3-ethylbenzthiazoline-6-sulfonic acid) (ABTS) to produce a radical cation (ABTS^+^), a soluble chromogen determined spectrophotometrically at 405 nm. In seminal plasma, TEAC was determined by comparing the optical density of each sample against a standard curve (range: 0–0.42 mM) of the water-soluble vitamin E analog. All the measures were carried out using reagents included in a commercial kit (cat. No. CS0790; Sigma-Aldrich, St. Louis, MO, USA).

For the detection carried out in spermatozoa, the pellet was resuspended in phosphate-buffered saline (PBS), pH 7.4, then diluted in the Assay Buffer (Sigma-Aldrich, Catalogue Number A3605) to obtain 1 million cells. The cells were lysed by sonication for 10 s (amplitude 60, 25 W; Vibracell Sonicator, VWR, Radnor, PA, USA), then centrifuged at 12,000× *g* for 15 min. The supernatant was recovered and applied in the TEAC assay.

### 2.7. Statistical Analysis

The data distribution was assessed using the D’Agostino and Pearson test. Data were reported as median and 95% confidence interval. Descriptive statistics and a correlation matrix (non-parametric Spearman correlation) for multiple variable analyses were performed on the group that included all data from both fertile and infertile subjects. False Discovery Rate (FDR) was calculated using the two-stage linear step-up procedure of Benjamini, Krieger and Yekutieli, by setting a q-value of 0.05 as significant.

Comparisons between the fertile and infertile groups were carried out using non-parametric (Mann–Whitney test) tests. The receiver operating characteristic (ROC) curve procedure was applied to evaluate the diagnostic performance of the parameters investigated in terms of the relationship between sensitivity and specificity, and then Youden’s Index (J) was calculated. The area under the curve (AUC) for each of the ROC curves was annotated with the 95% confidence interval (CI) by Wilson/Brown.

The statistical significance was defined as *p* < 0.05. The data analysis was carried out by the Graph-Pad Prism 8.4.2 statistical software package.

## 3. Results

In this study, 80 subjects were enrolled—18 fertile subjects and 62 infertile patients. Spermiogram was performed according to WHO guidelines [31,32] and sperm ultrastructure was analyzed by TEM; data was processed as reported by Baccetti and colleagues [23]. F_2_-IsoPs and RvD1 were measured in seminal plasma, TEAC in both spermatozoa and seminal plasma. All the variables were correlated by Spearman’s rank correlation coefficient in the whole population (Table 1). Semen parameters evaluated by light microscopy showed positive correlations with the fertility index and negative correlations with sperm necrosis detected by TEM. Apoptosis negatively correlated with sperm concentration, progressive motility and vitality, immaturity with sperm concentration and normal morphology (Table 1). Sperm concentration, progressive motility, normal morphology, vitality and fertility index negatively correlated with sperm TEAC, RvD1 level, and F_2_-IsoP concentration. Sperm TEAC showed positive correlations with sperm necrosis, apoptosis, and seminal RvD1 and F_2_-IsoP levels. RvD1 level positively correlated with seminal TEAC and F_2_-IsoPs. F_2_-IsoP levels correlated positively with sperm immaturity (Table 1).

Then, the participants in the study were divided according to their condition of fertility or infertility (Table 2). In the fertile group, the medians of all the seminal variables were higher than 25th centile [31]; the percentages of apoptosis, necrosis and immaturity were within the normal range (apoptosis 4.8%; necrosis 21.0%; immaturity 55.1%) and the fertility index was higher than 2 million, thresholds set for fertility thresholds reported by Baccetti and colleagues [23]. In the infertile group, the medians of sperm concentration, progressive motility, morphology, and vitality were over the fifth percentile [31]. The percentage of sperm apoptosis, necrosis, and immaturity were higher than normal values.

Comparing the variables between groups, conventional semen parameters and fertility index were significantly lower in the infertile group than those measured in fertile subjects (Table 2). The percentages of sperm apoptosis, necrosis, and immaturity were significantly increased in the infertile group with respect to the fertile group.

**Figure 2:**
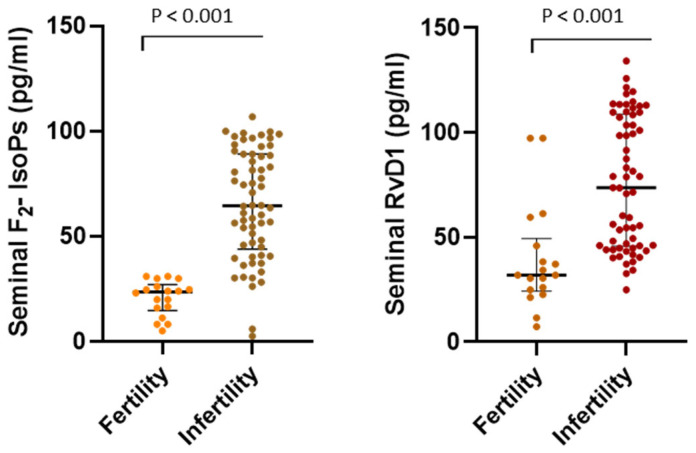
Data are reported as individual values; median and interquartile range are displayed. Statistical comparisons between groups were performed using non-parametric (Mann–Whitney test) tests. Statistical significance was set at *p* < 0.05.

**Figure 3:**
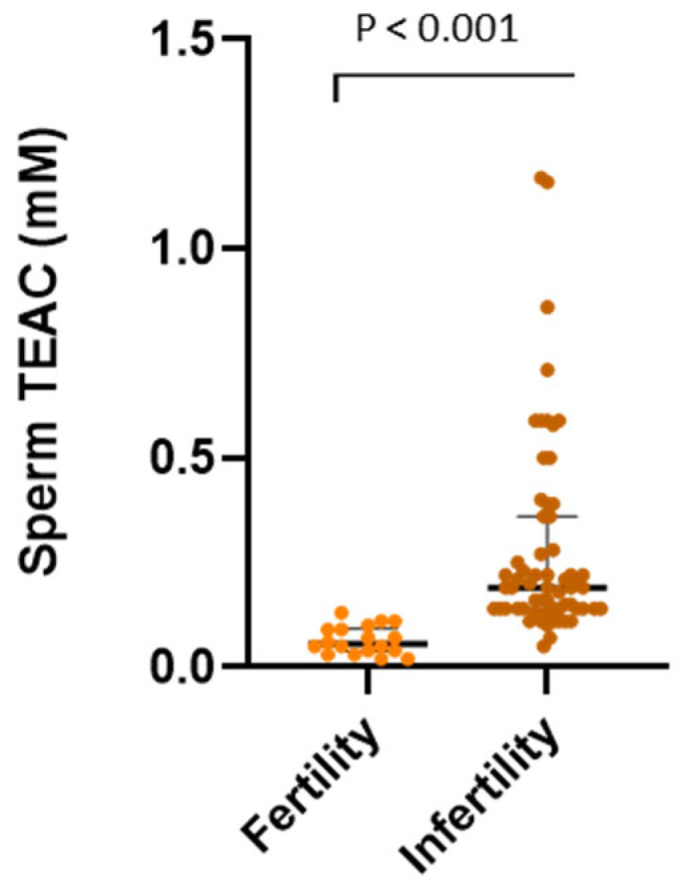
Data are reported as individual values; median and interquartile range are displayed. Statistical comparison between groups was performed using non-parametric Mann–Whitney test. Statistical significance was set at *p* < 0.05.

For F_2_-IsoPs, RvD1, and sperm TEAC, which are relevant parameters for the aim of the study and whose levels were significantly increased in seminal plasma of infertile men compared to fertile subjects (Table 2), data distribution is graphically displayed in Figure 2 (seminal F_2_-IsoPs and RvD1) and Figure 3 (sperm TEAC).

Seminal TEAC values were similar in the two groups analyzed (Table 2). To assess the relevance of seminal TEAC on seminal RvD1 and F_2_-IsoP levels, RvD1 and F_2_-IsoP values normalized to seminal TEAC values were calculated in fertile and infertile subjects. Both seminal RvD1/seminal TEAC and seminal F_2_-IsoPs/seminal TEAC ratios detected in the infertile group were higher than the fertile group (*p* < 0.001), as shown in Table 3.

**Figure 4:**
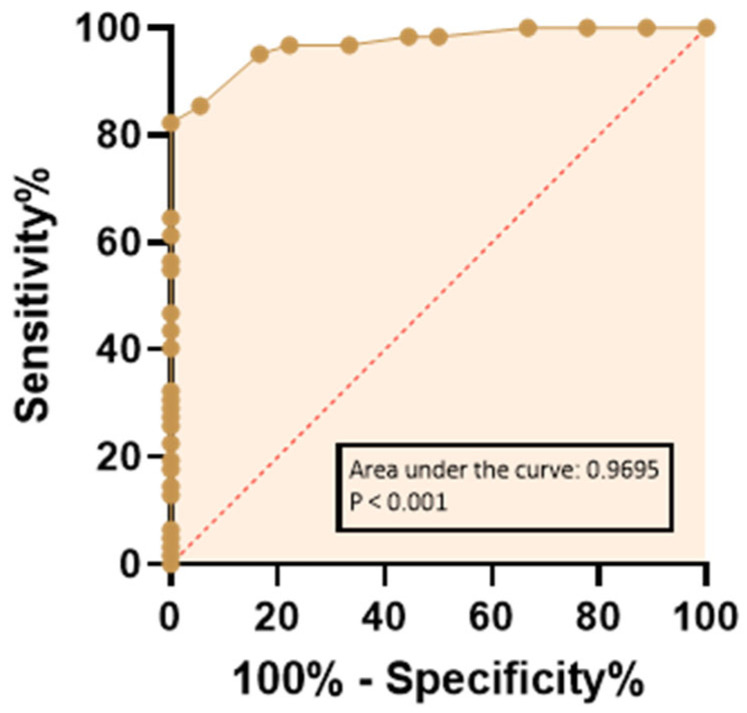
Prediction of infertility based on sperm TEAC values on the ROC curve. The dashed line indicates a completely random hypothesis. Controls (fertile group), n = 18; patients (infertile group), n = 62; missing controls, n = 0; missing patients, n = 0.

**Figure 5:**
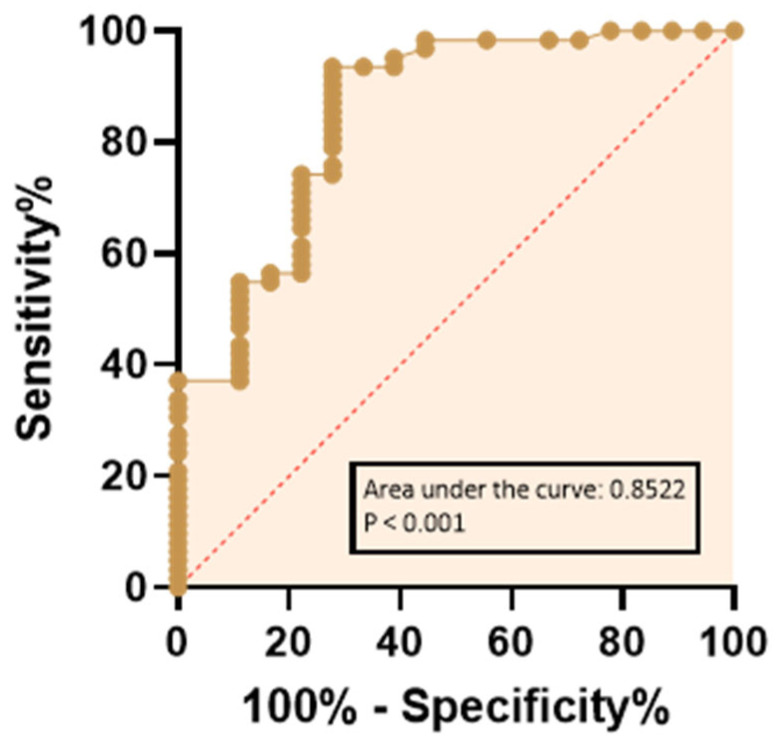
Prediction of infertility based on seminal RvD1 values defined on the ROC curve. The dashed line indicates a completely random hypothesis. Controls (fertile group), n = 18; patients (infertile group), n = 62; missing controls, n = 0; missing patients, n = 0.

Furthermore, sperm TEAC values were shown to discriminate between fertile and infertile subjects, as demonstrated by ROC curve analysis (area under the ROC curve: 0.97; 95% confidence interval: 0.94–1.0; *p* < 0.001, Figure 4). The highest J value indicated the optimal exploratory cut-off corresponding to 0.13 mM sperm TEAC. Interestingly, seminal RvD1 levels also accurately discriminated between fertile and infertile subjects, although with less accuracy than sperm TEAC (ROC curve analysis; area under the ROC curve: 0.85; 95% confidence interval: 0.74–0.96; *p* < 0.001, Figure 5). For RvD1, the highest J indicated the optimal exploratory cut-off as 38.26 pg/mL.

## 4. Discussion

Over recent decades, substantial evidence has highlighted the central role of OS in the etiology of male infertility. Conditions such as varicocele, genitourinary infections, and inflammatory disorders have been associated with increased production of ROS, resulting in enhanced LPO and subsequent impairment of sperm parameters such as sperm motility, viability, and DNA integrity, compromising male fertility potential. Notably, elevated LPO levels have also been observed in a substantial proportion of patients with idiopathic infertility, suggesting that OS may represent a common underlying mechanism even in the absence of identifiable clinical conditions [33,34]. Therefore, at least a portion of patients currently classified as having idiopathic infertility could today be reclassified as having infertility linked to oxidative causes. For this reason, it is important to study mediators of inflammation and markers of oxidative damage. In particular, attention should be given to their relationship with the antioxidant system of human semen, composed of seminal plasma and spermatozoa, both equipped with a complex antioxidant defence system that plays a crucial role in maintaining redox balance and protecting male reproductive function. Seminal plasma provides the primary antioxidant protection, acting as a rich reservoir of both enzymatic and non-enzymatic antioxidants; however, sperm cells themselves also possess intrinsic antioxidant capacity, although in limited amounts. Together, these systems act synergistically to neutralize ROS, prevent LPO of sperm membrane, and preserve sperm parameters [35].

The current state of the art underscores the pivotal role of oxidative imbalance in male infertility and highlights the importance of further investigating redox status as both a diagnostic marker and a potential therapeutic target. Among the available biomarkers, F_2_-IsoPs represent reliable indicators of OS in seminal plasma, and are considered more accurate than MDA, as F_2_-IsoPs are chemically stable end-products of LPO formed in situ through free radical-mediated oxidation of arachidonic acid, independent of enzymatic pathways [29,36]. The F_2_-IsoPs measured in seminal plasma, obviously, do not originate merely from spermatozoa or from cells present in the semen, such as leukocytes and immature germ cells, but also from cells located in various regions of the male reproductive tract, particularly from accessory glands such as the prostate and seminal vesicles, which produce a large amount of seminal plasma. Another lipid mediator considered in this study is RvD1, which, in human semen, was reported to increase with other markers of OS and inflammation [37].

This study aims to evaluate the interplay between antioxidant capacity in both seminal plasma and spermatozoa, and lipid mediators in determining the final extent of LPO in semen samples from infertile subjects, where oxidative damage to membrane fatty acids is considered relevant. The composition and concentration of fatty acids are fundamentally important in influencing male fertility [38,39], particularly regarding the amounts of different categories of PUFAs and the n-3/n-6 PUFA ratio [30,40]. In this study, parameters involved in the pathological processes of inflammation and LPO, as well as in antioxidant defence, were examined to evaluate how these events are interconnected in cases of male infertility. TEAC measures the overall ability of a biological sample to neutralize free radicals. More specifically, it assesses how effectively the antioxidants present in the sample can scavenge a defined radical species. Results are expressed relative to Trolox, a water-soluble analogue of vitamin E, which is used as a standard reference compound. For this reason, in selecting patients, we excluded subjects who had taken antioxidant supplements including vitamin E, as this is a common practice frequently recommended by clinicians in cases of male infertility [41,42,43,44].

The observation that TEAC evaluated in spermatozoa (not in seminal plasma) differs significantly between fertile and infertile subjects, and is increased in the infertile group, is consistent with the hypothesis that spermatozoa in infertile subjects may show a compensatory antioxidant response to oxidative imbalance.

This observation is also supported by the positive correlations observed between sperm TEAC and F_2_-IsoPs and by the negative correlation between sperm TEAC and fertility index. The oxidative damages affecting spermatozoa of infertile patients are clearly shown by ultrastructural TEM analysis mathematically elaborated by the increased percentage of sperm apoptosis and necrosis, which likely reflect a possible inflammatory condition or resolution-related processes, highlighted by RvD1 levels, and a persistent OS condition, suggested by F_2_-IsoP concentration [27,28,45].

The positive correlation between sperm TEAC with F_2_-IsoP levels further indicated that lipid oxidative damage may be modulated by the antioxidant defences that act to counteract it. The normalization of the seminal F_2_-IsoP amounts with respect to seminal TEAC values allowed us to identify a parameter that is significantly different between fertile and infertile subjects (increased in the infertile group), and this is due in particular to the F_2_-IsoP concentration, which is clearly increased in the infertile groups. As part of the defence against LPO, the seminal lipid mediator RvD1, which is involved in inflammation resolution, and both sperm and seminal TEAC are positively correlated in the studied population. This finding aligns with current understanding that antioxidants not only neutralize ROS but also modulate inflammatory pathways, thereby mitigating OS and chronic inflammation [46,47]. To this aim, antioxidant strategies are focused on balancing pro- and anti-inflammatory signals [46]. However, in our study, antioxidant capacity appears to be only one of the factors, as well as autophagy [48,49] and omega-3 PUFA availability [50], regulating the production of pro-resolving lipid mediators, as significant differences between fertile and infertile subjects persist even after normalization for seminal TEAC.

**Figure 6:**
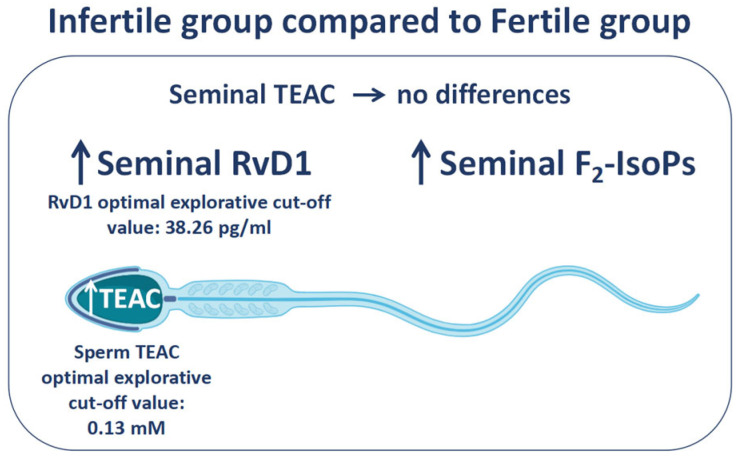
The figure shows the values of Resolvin D1 (RvD1) and F_2_-Isoprostanes (F_2_-IsoPs) measured in seminal plasma and Trolox equivalent antioxidant capacity (TEAC) dosed in both seminal plasma and spermatozoa. The indices, except for seminal TEAC, resulted higher in the infertile group compared to fertile group. The exploratory optimal cut-off values of seminal RvD1 (38.26 pg/mL) and sperm TEAC (0.13 mM) are reported.

The key focus of the study was the identification of two exploratory indices capable of discriminating between fertile and infertile subjects, namely TEAC in spermatozoa (cut -off: 0.13 mM) and RvD1 levels (cut-off: 38.26 pg/mL) measured in seminal plasma (Figure 6), in addition to previously proposed markers, such as the fertility index derived from mathematically elaborated TEM analysis and F_2_-IsoPs [18,23]. It is well established that conventional semen analysis does not provide a comprehensive parameter capable of clearly distinguishing between fertility and infertility; therefore, the identification of possible biomarkers for the diagnosis of male infertility remains crucial. In this context, particular attention has been devoted to OS markers, given that OS represents a major cause of impaired sperm function and male infertility [51]. From a different perspective, but of comparable importance, there is the need to better understand the role of sperm evaluation in predicting the outcomes of ART [52].

Given the relevance of the TEAC data presented here, it is important to note that measurement of total antioxidant capacity (TAC) remains one of the most widely used methods to assess a sample’s oxidant-buffering potential. In biological samples, TAC is recognized as a valuable indicator of OS-related health status, and its use for point-of-care monitoring has been proposed [53]. Interestingly, TAC assays are also frequently used to identify the antioxidant capacity in male infertility [8]. Here, the TEAC test was applied as it is the most frequently used indirect assay to measure antioxidant capacity. Nevertheless, several methods for assessing the antioxidant capacity of human seminal plasma have been proposed [54]. Among these, the ferric reducing antioxidant power (FRAP) assay has also been applied to seminal plasma analysis [55]. Therefore, when comparing findings across studies, careful consideration must be given to the underlying principles of the analytical methods used, commonly grouped under the term “TAC”.

Our results show that sperm antioxidant activity could be a useful parameter to discriminate between fertile and infertile subjects, but this defensive potential cannot completely hinder F_2_-IsoP production detected in seminal plasma.

This study clearly has some limitations. The most important ones include the need to increase the sample size and to further investigate the behaviour of lipid mediators and TEAC under specific conditions known to affect fertility, such as varicocele, leukocytospermia, and urogenital infections. Another limitation is that F_2_-IsoPs were not measured in spermatozoa, although they are known to respond to oxidative damage by increasing their antioxidant capacity.

## 5. Conclusions

This study highlights two key findings. First, spermatozoa have been observed to exhibit higher antioxidant capacity in the presence of an OS environment; this data suggests an association in which antioxidant defences may vary in relation to the surrounding seminal plasma.

Second and equally important, TEAC measured in spermatozoa, and to a lesser extent RvD1 measured in seminal plasma, emerged as candidate biomarkers for identifying infertile patients. These findings certainly warrant further investigation and deeper exploration.

## Figures and Tables

**Table 1:** Spearman correlation coefficients (r) between investigated variables.

	Volume (mL)	Sperm Concentration ×10^6^/mL	Progressive Motility (%)	Normal Morphology (%)	Vitality (%)	Necrosis (%)	Immaturity (%)	Apoptosis (%)	Fertility Index (n°)	Seminal RvD1 (pg/mL)	Seminal F_2_-IsoPs (pg/mL)	Seminal TEAC (mM)	Sperm TEAC (mM)
Volume (mL)	1												
Sperm concentration ×10^6^/mL	−0.01	1											
Progressive motility (%)	0.05	0.42 ***	1										
Normal morphology (%)	0.16	0.70 ***	0.33 **	1									
Vitality (%)	0.18	0.40 ***	0.42 ***	0.54 ***	1								
Necrosis (%)	−0.16	−0.32 **	−0.33 **	−0.42 ***	−0.69 ***	1							
Immaturity (%)	−0.05	−0.24 *	0.07	−0.23 *	0.05	−0.16	1						
Apoptosis (%)	−0.01	−0.28 **	−0.38 ***	−0.15	−0.30 **	0.38 ***	0.02	1					
Fertility index (n°)	0.29 **	0.41 ***	0.37 ***	0.47 ***	0.49 ***	−0.4 ***	−0.22 *	−0.29 **	1				
Seminal RvD1 (pg/mL)	−0.22	−0.22 *	−0.38 ***	−0.31 **	−0.33 **	0.39 ***	0.22 *	0.29 **	−0.34 **	1			
Seminal F_2_-IsoPs (pg/mL)	−0.11 **	−0.28 ***	−0.38 *	−0.60 ***	−0.37 **	0.26 *	0.44 ***	0.13	−0.31 **	0.29 **	1		
Seminal TEAC (mM)	0.05	0.08	−0.18	0.02	−0.18	0.11	0.00	−0.11	−0.16	0.44 ***	−0.03	1	
Sperm TEAC (mM)	−0.17	−0.51 ***	−0.4 ***	−0.51 ***	−0.46 ***	0.47 ***	0.18	0.35 **	−0.43 ***	0.41 ***	0.636 ***	0.04	1

Statistical significance, adjusted *p*-value that accounts for multiple comparisons, controlling FDR: *p* ≤ 0.05 *; *p* ≤ 0.01 **; *p* ≤ 0.001 ***. Sample: *n* = 80.

**Table 2:** Variables evaluated in fertile and infertile subjects.

Variables	Median (25th–75th Percentiles)		Statistics
	Fertile Group	Infertile Group	
Volume (mL)	4.50 (3.50–5.00)	3.50 (2.78–4.00)	*p* < 0.01
Sperm concentration ×10^6^/mL	98.00 (57.00–123.70)	23.50 (12.28–54.50)	*p* < 0.001
Progressive motility (%)	53.50 (47.25–69.50)	35.00 (21.00–52.50)	*p* < 0.01
Normal morphology (%)	15.50 (14.00–20.00)	6.00 (3.00–9.00)	*p* < 0.001
Vitality (%)	85.00 (83.00–88.25)	70.00 (51.00–75.00)	*p* < 0.001
Apoptosis (%)	4.93 (4.06–7.27)	7.89 (5.26–11.12)	*p* < 0.01
Necrosis (%)	23.21 (21.57–33.00)	36.22 (30.78–47.99)	*p* < 0.001
Immaturity (%)	51.00 (46.36–54.59)	65.34 (53.78–71.35)	*p* < 0.001
Fertility index (n°)	3,074,355 (2,133,507–4,440,662)	457,187 (45,342–876,224)	*p* < 0.001
Seminal F_2_-IsoPs (pg/mL)	23.50 (14.73–27.05)	64.50 (43.90–89.10)	*p* < 0.001
Seminal RvD1 (pg/mL)	31.88 (24.35–49.36)	73.65 (45.67–108.80)	*p* < 0.001
Seminal TEAC (mM)	2.07 (1.72–2.28)	2.13 (1.96–2.36)	NS
Sperm TEAC (mM)	0.05 (0.04–0.09)	2.13 (1.96–2.36)	*p* < 0.001

Data are reported as median (25th–75th percentile). Statistical significance was set at *p* < 0.05. NS: not significant.

**Table 3:** Seminal RvD1 or seminal F_2_-IsoPs/seminal TEAC ratio in fertile and infertile subjects.

Seminal RvD1 and Seminal F_2_-IsoPs to Seminal TEAC Ratio	Median (25th–75th Percentiles)		Statistics
	Fertile Group	Infertile Group	
Seminal RvD1/ seminal TEAC ratio	18.20 (12.31–24.36)	36.02 (24.18–47.67)	*p* < 0.001
Seminal F_2_-IsoPs/ seminal TEAC ratio	10.80 (7.05–15.09)	32.10 (20.51–44.17)	*p* < 0.001

Data are reported as median (25th–75th percentile) and statistically compared using the non-parametric test (Mann–Whitney test). Statistical significance was set at *p* < 0.05.

## Data Availability

The data generated and analyzed during this study are included in this published article and are available from the corresponding author. The data are not publicly available due to the privacy of the patients.
